# DEI co-mentoring circles for clinical research professionals: A pilot project and toolkit

**DOI:** 10.1017/cts.2022.517

**Published:** 2022-12-22

**Authors:** H. Robert Kolb, Tiffany Danielle Pineda, Angela Sow, Michael Hinton, Martin Noguera, Tatiana Ramirez-Hiller, Gailine McCaslin, Carolynn Thomas Jones

**Affiliations:** 1 University of Florida, Clinical Translational Science Institute, College of Medicine, Gainesville, FL, USA; 2 Ohio State University, Center for Clinical Translational Science, Columbus, OH, USA; 3 Ohio State University, College of Nursing, Columbus, OH, USA; 4 Ohio State University, Comprehensive Cancer Center, Columbus, OH, USA

**Keywords:** Mentoring, co-mentoring circles, peer mentoring, clinical research professionals, DEI

## Abstract

**Background:**

There have been a number of federal policies and guidance’s impacting diversity, equity, inclusion, and accessibility (DEI) in clinical research. While these are needed, they have not diminished the gaps related to clinical trial recruitment, research professional’s capacity for cultural competence, and clinical research professional role development. Mentoring and co-mentoring circles have traditionally been used in Medicine, but until now had not been used for workforce development of clinical research professionals (CRPs).

**Materials/Methods:**

We designed a six-session, monthly co-mentoring circle to take place at two academic medical centers to pilot an interinstitutional co-mentoring circle centered on storytelling videos of Black Voices in Clinical Research. This provided a DEI framework for discussions on role experiences, cultural competence, and role progression.

**Results:**

Seven CRPs completed the DRC pilot. The participants positively evaluated the experience and made recommendations for future iterations. *Discussion:* Co-mentoring circles can be useful tools to connect CRPs across complex research medical centers and provide support that may have a positive impact on role satisfaction and retention.

**Conclusion:**

This framework for developing co-mentoring circles can serve as a toolkit for future CRP co-mentoring circles within and across institutions for workforce development. The Black Voices in Clinical Research storytelling videos provide a rich foundation for future discussion on DEI issues for CRPs and collaborating with participants.

## Introduction

Since 2017, there has been an increasing series of policies and initiatives that address diversity, equity, inclusion, and accessibility (DEI) issues for clinical research workforce, workforce education and training, and participant recruitment [1–5] (see Table 1). However, while varying levels of disparity exist in study design, public trust, and study recruitment, DEI disparities also exist in clinical research professional workforce development, especially mentoring [6].

**Table 1.** Evolution of Diversity Policies at NIH and FDA (2017–2022)

• 2017 – NIH Policy and Guidelines on the Inclusion of Women and Minorities as Subjects in Clinical Research [3] • 2019 – Notice of NIH’s Interest in Diversity [5] • 2020 – FDA’s Enhancing the Diversity of Clinical Trial Populations – Eligibility Criteria, Enrollment Practices, and Trial Designs Guidance for Industry [4] • 2022 - NIH – Wide Strategic Plan for DEI [1] • 2022 – NIH UNITE – Ending Structural Racism [2]

The 1993 National Institutes of Health (NIH) Revitalization Act required NIH-funded clinical trials to include women and minorities as participants [7], but those inclusion goals fell short. A familiar aphorism, “the more things change, the more they stay the same,” captures the nature of this shortcoming. The persistent lack of diverse enrollment in research studies carries critical implications when research findings are translated and presented to the general population [8–10]. The COVID-19 pandemic, compounded with the murder of George Floyd, unveiled global health inequity issues that fueled awareness, education, policies, and a new DEI perspective on clinical research [11–13].

### DEI Related Issues in the Clinical Research Professional Environment

Clinical research professionals (CRPs) regularly face significant barriers to successful enrollment of women, children, underrepresented minorities, LGBTQIA+, and those with diverse abilities [14–18]. Educating the CRP workforce on DEI skills such as cultural awareness, unconscious bias, micro-aggressions, and sensitivity trainings can greatly assist the clinical research team as well as recruitment, care, and retention of clinical trial participants. For instance, CRPs who have been equipped to interact with diverse populations are able to create a sense of belonging and experience favorable interactions, exhibiting a notable level of respect, which will directly impact health equity [19,20]. Moreover, recent studies confirm that diversity of the workforce directly correlates with a more diverse participant population [21].

To ensure robust cultural competency in the workforce, it is important to have a working environment that encourages CRPs to become competent communicators and actors in the intersectional areas of diversity and culture. Cultivating a culturally competent workforce begins with valuing, supporting, and addressing the unique challenges experienced by each individual. Though all new professionals may encounter obstacles to gain employment in the field, staff from diverse backgrounds more often encounter unique barriers related to DEI challenges. Additionally, while there may be varying levels of generic support structures in place to help advance new careers, diverse staff encountering DEI specific challenges typically do not have focused supportive structures in place to further encourage their careers [6].

### DEI Participant Initiatives and Black Voices in Research

Several initiatives have come to fruition that address health inequities and aid in improving awareness and best practices. The Society for Clinical Research Sites has identified a Diversity Site Assessment Tool that can aid in awareness and benchmarking [14,22]. Beyond targeting patient diversity recruitment awareness, the incorporation of health equity and diversity during the design of clinical research studies is essential. To address these design challenges, the Multi-Regional Clinical Trials Center produced the Equity by Design (EbD) Metrics Framework, Version 1.1, which provides an evidence-based approach to guide researchers in applying DEI policies in study design, training, and outreach [23]. The University of Florida (UF) Clinical and Translational Science Institute (CTSI) formed a Diversity and Cultural Competence Council (DC^3^) that provides training to improve DEI. Using an innovative approach, the UF DC^3^ developed a series of storytelling videos entitled: *Black Voices in Research* [24]. Storytellers include eight black faculty researchers, graduate research assistants, administrators, CRP staff, and community members, who were given storytelling coaching to efficiently articulate their experiences leading to a career in research and how they personally navigated structural, systemic, and societal barriers in their journeys while harnessing personal character strengths and resilience. This work led to publicly available videos and toolkits for use in DEI training at UF and for other CTSA hubs.

### Mentoring and Co-Mentoring for Professional Development

Co-mentoring is the merging of collaboration and mentoring [25]. Mentoring has many definitions but in its simplest form, it is the intentional relationship between two individuals for the growth of one (or both) participants [26]. Traditionally in medicine and translational research, mentoring workforce development goals is met through interactions between a senior mentor and the student or early career individual [27]. Advancements in mentoring structures have positively contributed to meeting researchers’ workforce development needs, especially addressing disparities in women and under-represented minority (URM) faculty and researchers [28,29]. Reverse mentoring is an alternative strategy that offers mentees an opportunity to provide information to the mentor (e.g., DEI knowledge, informatics, and technology knowledge) [30]. Yet another alternative strategy is peer-to-peer mentoring.

**Fig. 1.**
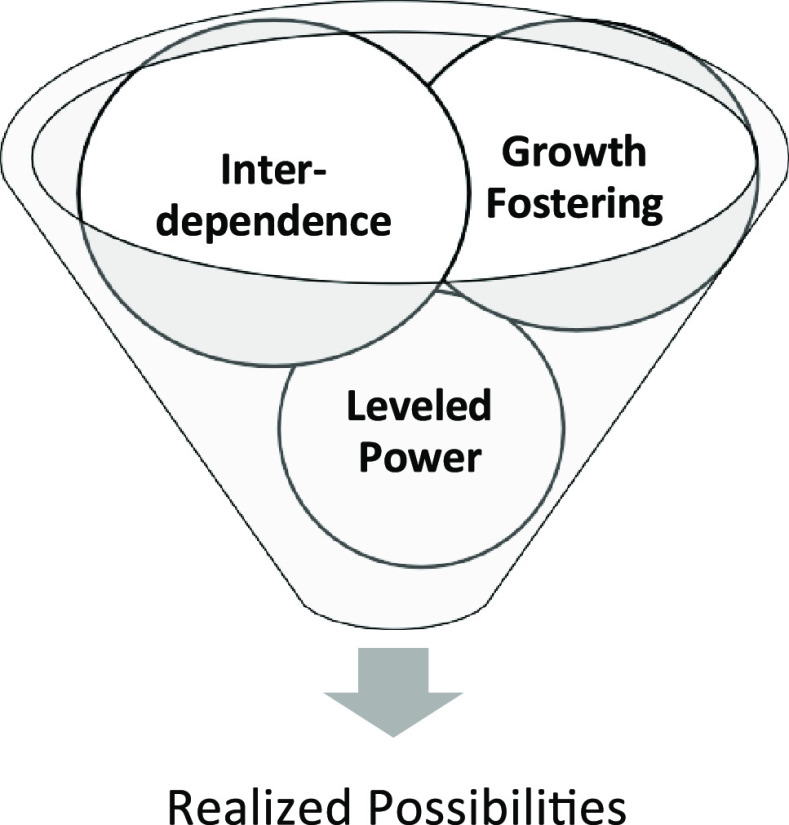
Relational-Cultural Elements in Co-Mentoring.

Co-mentoring circles are a type of group mentoring usually involving a small group of mentees (5 to 7 members), organized around a facilitator, and focused on topics supporting learning and development within an organization. Fostering these circles promises to create a more egalitarian mentoring culture, especially for CRPs who often lack any form of mentoring in their careers outside of their direct supervisors. CRP staff collaborate and engage in co-creating the circle. Co-mentoring circles draw on a model of relational mentoring which allows a “full range of processes, mechanisms, and outcomes of developing relationships” (p. 374) based on earlier work on Relational-Cultural Theory (RCT) [31]. The foundational concept of RCT is that the change comes out of connection. Elements of RCT include mutual empowerment and empathy that fosters growth, allows interdependence, and levels power structures through reflection and checking in, leading to realized possibilities (Fig. 1) [32].

## Methods

To explore an alternative workforce development strategy to address DEI, we created and piloted a collaborative DEI co-mentoring circle for CRPs working at two CTSA Hubs, aiming to create a model which stimulates reflection as well as sharing and solution finding for DEI issues of the workforce in clinical research management work. We used the DC^3^ Black Voices in Clinical Research [33] as a basis for discussions during the six-session series of co-mentoring meetings.

### Process

**Fig. 2.**
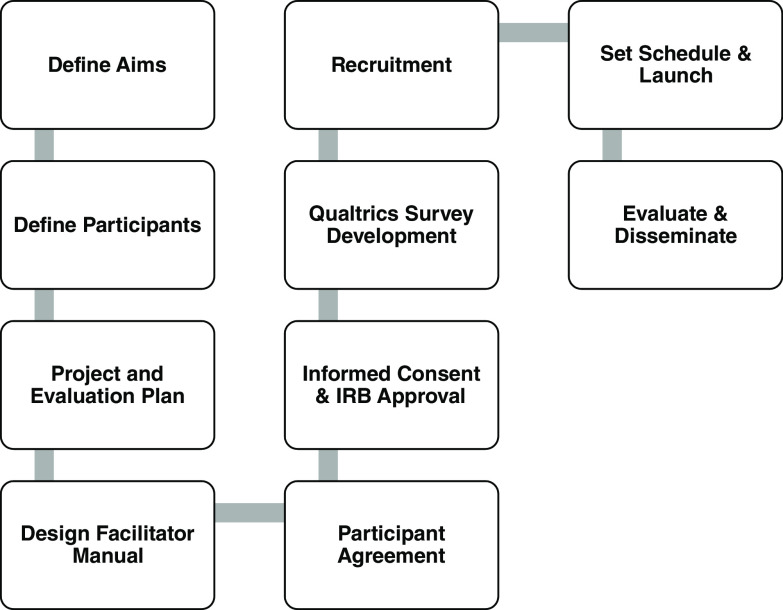
Process of Developing the Co-Mentoring Circle.

We conceptualized the collaborative co-mentoring circle approach for DEI training for CRPs by assembling a team from both University of Florida CTSI and the Ohio State University Center for Clinical Translational Science (CCTS). Our steps to developing the co-mentoring circle is illustrated in Fig. 2. Our aims were to 1) develop a safe-space co-mentoring circle that used the DC^3^ Black Voices in Clinical Research, 2) stimulate reflection and discussion on issues resonated from the videos, and 3) discuss the participant’s own stories about entering and progressing in clinical research professional roles.

We developed a project charter, activity plan, survey instruments, and recruitment plan. We also developed a facilitator guide with scripts and pre-work assignments. Finally, we drafted a co-mentoring circle agreement for participants. Our circle’s agreement included common commitments for co-mentoring circles (Table 2). Participants were invited to edit and add to these commitments.

**Table 2.** Elements of a co-mentoring circle agreement

General commitment	Inter-relational commitment
• Meet regularly – maintain communication • Keep camera on during zoom meetings • Be present to the circle during meetings • Review progress and actively share • Provide guidance, support, encouragement • Provide feedback regarding process experience	• Confidentiality of discussions, • Attentive listening, • Appreciation – *No put-downs,* • Express mutual respect, • Acknowledge others right to pass or be silent, • A capacity to create community.

Because of the reflective nature of the co-mentoring circles, individuals were encouraged to reflect and journal about topics raised during the meetings.

We designed the meeting plan to allow rotation of facilitation by different facilitators from University of Florida (UF) and The Ohio State University (OSU) to avoid the appearance of a single “lead.” At project launch, we provided facilitators with instruction on how to lead a co-mentoring circle session to insure participant engagement, safety, and prompt discussion. Using focus group approaches, facilitators used a script to start the conversations and to prompt discussion without “leading,” and encouraged to share as a member of the circle without inserting a personal agenda or authority. This enabled a safe space for participants to view themselves as co-mentors who were co-creating the process through sharing and reflection. We held the sessions every four weeks, via Zoom, for six sessions.

### Evaluation Plan

Our intake survey included quantitative and qualitative responses including demographics, experience level in clinical research, mentoring experiences, how they entered and progressed from their first role in clinical research, satisfaction in role progression, institutional factors, and any contemplations about leaving the institution. Descriptive statistics were planned to evaluate the initial intake survey and served to set stage to understand the group.

The mid-point and exit surveys served to evaluate participant experiences in the process and satisfaction with the co-mentoring circle. Descriptive statistics were planned to evaluate these two surveys. The final session also served as an evaluation dialogue with participants about what worked well? What did not? What do they suggest for future co-mentoring experiences? What expectations were or were not met?

## Results

### Participant Demographics

In total, we had ten co-mentoring circle participants including one facilitator from UF and two from OSU. Seven of the participants, including facilitators, completed the intake survey. None of the participants had ever participated in a co-mentoring circle or a traditional mentoring experience. The demographic constitution of our group was primarily biracial, multiracial, or Black/African American. Half of the participants were Hispanic or Latino, and the majority of participants were female. In this cohort, there were varying levels of education and experience from bachelor’s degrees to Ph.D. candidates and levels of experience ranging from five years’ experience to more than two decades. As is common in most institutions, there were varying job titles.

### Participant Workforce Development Issues

We asked about ease of getting a first job in clinical research. Three individuals rated it difficult (1) or very difficult (2) to get their foot in the door for their first job. Qualitatively, those who did not have difficulty shared how they got their first job:

- *“A friend told me about a job. I got the job through word of mouth.”*
- *“The University posted an ad and I applied and got the job.”*
- *“I wanted to move to Gainesville FL, so I searched for jobs online and applied.”*

The respondents did recognize the institutions dedication to improve CRP diversity; however, for the other questions on role progression, performance review, workforce DEI, results were skewed to dissatisfied and very dissatisfied. Regarding the availability of CRP mentors, responses leaned equally from neutral to very dissatisfied. Table 4 displays survey responses related to job satisfaction at the time of entering the co-mentoring circle.

**Table 3.** Outline of sessions for the pilot DEI co-mentoring circle

Sessions/Steps	Topics
Pre-session	• Emails to volunteers • Informed consent • Pre-meeting Qualtrics^xm^ survey
One Form the Circle	• Kickoff • Creating the community (forming the circle) – Introductions • Discussion on Project AIMS and sharing intake survey results • Describe your entrance to the clinical research profession • Describe your experiences with being mentored, being a mentor • Assignment: For the next meeting, preview Episode One of Black Voices in Clinical Research, Reflect/make notes
Two Begin the Conversation	• Discussion about Episode One • Describe your feelings and thoughts that personally resonated with you from the stories shared in Episode One • Did any experiences stand out? • What about personal attributes of the storyteller? • Assignment: For the next meeting, preview Episode Two of Black Voices in Clinical Research, Reflect/make notes
Three Expand the Conversation	• Check in for Session Three (feelings, concerns) • Discussion about Episode Two • Describe your feelings and thoughts that personally resonated with you from the stories shared in Episode Two • Did any experiences stand out? • What about personal attributes of the storyteller? • What are your personal experiences with entering the field and role progression?
Four Expand the Conversation	• Check in for Session Four (feelings, concerns) • What structural racism issues inhibit CRP workforce diversity and inclusion? • How can we assist novices to progress? • How can we create a more diverse CRP workforce? • Complete Midpoint Evaluation Survey (Qualtrics ^xm^)
Five Expand the Conversation	• Check in for Session Five (feelings, concerns) • What community outreach can diversify the CRP workforce? • What are possible innovative career pathways that would improve progression? • Solution-finding discussion and sharing
Six Wrap up and Future Goals	• Check in for Session Six (feelings, concerns) • Continue the discussion, list take home messages • Identify future goals and possible continuations or collaborations • Discuss what each person wants and needs for their career • Complete Final Evaluation Survey (Qualtrics ^xm^)

CRP, clinical research professional.

**Table 4.** Job satisfaction results from the initial intake survey (n = 7)

Satisfaction Factor	Very Satisfied	Satisfied	Neutral	Dissatisfied	Very Dissatisfied
Role Progression Planning	0	1 (14.3%)	1 (14.3%)	4 (57.1%)	1 (14.3%)
Performance Review Process	1 (14%)	1 (14.3%)	2 (28.5%)	4 (57.1%)	0
CRP Workforce DEI	0	1 (14.3%)	1 (14%)	4 (57.1%)	1 (14.3%)
Institution Dedication to Improve CRP DEI	0	1 (14.3%)	3 (43%)	3 (43%)	0
Availability of CRP Mentors	0	1 (14.3%)	2 (28.5%)	2 (28.5%)	2 (28.5%)
Institutions Culture of Teamwork and Collaboration	1	3 (43%)	1 (14.3%)	4 (57.1)	0

DEI, diversity, equity, and inclusion; CRP, clinical research professional.

We also asked participants if they had thoughts about leaving the institution. Only one stated “Never,” four indicated “Sometimes,” and two indicated “Rarely.”

### Midpoint Evaluation

At the end of week four, we solicited feedback from participants at midpoint; however, only five individuals completed the midpoint survey and there were no selections for “moderate amount of the time,” “not very much” or “never” (Table 5).

**Table 5.** Midpoint evaluation results (n = 5)

Satisfaction results	All of the time	Great deal of the time
Was the co-mentoring circle accessible to you?	4 (80%)	1 (20%)
Did you have time for sharing?	5 (100%)	0
Did you feel heard?	4 (80%)	1 (20%)
Were the circles collegial?	4 (80%)	1 (20%)
Were the circles respectful?	5 (100%)	0
Were sessions well organized?	4 (80%)	1 (20%)
Were sessions well facilitated?	4 (80%)	1 (20%)

### Final Survey Results

In the final session, we had an open discussion evaluating the co-mentoring circle. The participants valued the videos as a “blueprint” and jumping off point for discussion.

- *“The videos were insightful, emotional and motivating”*
- *“The videos motivated me to assist in the efforts of overcoming systemic racial prejudice in the academic institution environment as well in my direct community”*
- *“I identified with the DC^3^ Black Voices in Research speakers, and it made me feel less isolated and I learned of a community I never knew existed.”*
- *“I feel more empowered to further concentrate my efforts, however I can, in aiding mentees of underrepresented, multicultural, and first-generation populations in having a successful and enlightening college experience, free of racism, bias, discrimination and prejudice”*

Collectively, participants stated that the emotional benefits of the co-mentoring circle included appreciation for “being heard,” “gaining inter-institutional perspectives”; “helped with sanity and mental health, emotional awareness, and “experiencing empathy, empowerment and hope.” Another participant stated that the DC^3^ stories moved them to understand the importance of wellness and community, stating “I submitted and was funded to study the impact on the wellness and sense of community of Black faculty, staff and students at my institution through West African dance.”

The participants wondered if six sessions were sufficient, although they agreed that the sessions should meet no more frequently than monthly, given their busy schedules. They thought future sessions should transition at session six and then use the circle to form actionable outcomes, such as setting and achieving attainable goals. They debated the size of the group, ten was almost too large and sharing may become limited, but that attrition reduced the ultimate number to seven. Many of the participants wanted to stay in touch after the sessions concluded and continue the support for one another in professional growth. Participants also expressed a willingness to facilitate future co-mentoring circles. Moreover, participants had several DEI-related suggestions to aid institutions to improve a culture of diversity and inclusion for clinical research professionals (Table 6).

**Table 6.** Suggestions for improving institutional diversity and inclusion

• Create and cultivate an environment that is inclusive, welcoming, and nurturing. • Be intentional in leveraging voices from under-represented minority staff when evaluating core values, strategic planning, policy development, leadership development, and community engagement. • Call upon underrepresented minority staff when social justice matters arise. • Include URM staff into the fabric of how we do business and service our internal, local, and national stakeholders. • Expand awareness of the community and culture to ensure that diverse peoples are welcomed. • Include DEI activities in faculty promotion and in staff advancement. • Consistently evaluate institutional culture, avoid tokenism (simply hiring diverse people). • Focus on retention of diverse employees. Conduct meaningful exit and “stay” interviews to identify factors associated with prejudice, incivility, unjust practices. • Compensate diverse people for the work they do for DEI efforts as representatives of the university’s efforts, this will mitigate the perception of tokenism. • Have actionable and periodic assessments related to the exemplification of mission statements and Native American land use acknowledgements.

DEI, diversity, equity, and inclusion; URM, under-represented minority.

## Discussion

Several publications exist to describe co-mentoring circles for clinical research scientists and women faculty [28,29,34]. Here, we report our pilot co-mentoring circle for DEI within CRPs as an innovative approach to address the urgent need to diversify and support the CRP workforce especially at Academic Medical Centers that are suffering from unprecedented staff turnover rates [35]. Co-mentoring circles have the potential to mitigate dissatisfaction and disconnections by fostering new ways to self-identify as a CRP leader to ultimately transform the experience into connection and future co-mentoring circles. Co-mentoring circles could also be used during the onboarding process to support staff transitioning to new CRP roles. This transformative process of turning disconnection into connection is at the core of RCT [36]. The participants in the co mentoring circles are engaged in deliberate relational work with the committed intention to feel like true partners in clinical research through connections that help to break down siloes.

Co-mentoring circles can lessen disconnection by-products such as feeling unsafe, violated, stuck, or even disappointed [37]. Pre-COVID-19 job satisfaction of CRPs revealed key factors in job satisfaction and retention including 1) understanding of the CRP role, 2) collaborative relationships with the PI, and 3) understanding of protocol development [38]. However, publications related to CRP mentoring, CRP relationships, and team science are lacking. Participating in a co-mentoring circle was a new territory for the CRPs, who expressed the value of the experience and desire for future co-mentoring circles. Additionally, it served to give voice to their experiences entering and navigating the field of clinical research and stimulated recommendations for bettering institutional goals to address diversity and inclusion.

## Conclusion

This project offers practical methods and lessons learned from forming co-mentoring circles, based on our pilot offering of a co-mentoring circle in addressing DEI disparities for CRPs working at two CTSA hubs. The DC^3^ Black Voices in Clinical Research offered a meaningful platform for stimulating conversation, reflection, and motivating ideas and action. In offering DEI co-mentoring support, we recommend longer sessions (over the course of a year) with incorporation of role progression support and goal setting. This model should be considered for other types of CRP co-mentoring circles, both locally and across institutions, on a variety of topics such as publication development, onboarding groups, leadership, mentoring, and CRP team-science training.
